# Geometric factors influencing the diet of vertebrate predators in marine and terrestrial environments

**DOI:** 10.1111/ele.12375

**Published:** 2014-09-30

**Authors:** Chris Carbone, Daryl Codron, Conrad Scofield, Marcus Clauss, Jon Bielby, Brian Enquist

**Affiliations:** 1 Institute of Zoology, Zoological Society of LondonRegent's Park, London, NW1 4RY, UK; 2 National Museum, Florisbad Quaternary ResearchPO Box 266, Bloemfontein, 9301, South Africa; 3 Clinic for Zoo Animals, Exotic Pets and Wildlife, Vetsuisse Faculty, University of ZurichWinterthurerstr. 260, CH-8057, Zurich, Switzerland

**Keywords:** Body size, elasmobranchs, energetics, macroecology, mammals, marine predators, predator–prey relationships, scaling, snakes

## Abstract

Predator–prey relationships are vital to ecosystem function and there is a need for greater predictive understanding of these interactions. We develop a geometric foraging model predicting minimum prey size scaling in marine and terrestrial vertebrate predators taking into account habitat dimensionality and biological traits. Our model predicts positive predator–prey size relationships on land but negative relationships in the sea. To test the model, we compiled data on diets of 794 predators (mammals, snakes, sharks and rays). Consistent with predictions, both terrestrial endotherm and ectotherm predators have significantly positive predator–prey size relationships. Marine predators, however, exhibit greater variation. Some of the largest predators specialise on small invertebrates while others are large vertebrate specialists. Prey–predator mass ratios were generally higher for ectothermic than endothermic predators, although dietary patterns were similar. Model-based simulations of predator–prey relationships were consistent with observed relationships, suggesting that our approach provides insights into both trends and diversity in predator–prey interactions.

## Introduction

Predator–prey relationships represent a fundamental component of community structure and have a major influence on ecosystem function and stability (Heithaus 2001; Blanchard *et al*. 2009). Terrestrial and marine ecosystems are the major biospheres of our planet, and while there are some similarities [e.g. body mass and maximum density (Belgrano *et al*. 2002)”, there are substantial differences in trophic complexity and predator–prey relationships which are still not fully understood (Cohen 1994).

Differences between predator–prey relationships in marine and terrestrial systems may, in part, be explained by characteristics of the physical environment. On land, movement and mobility are greatly affected by gravity (Schmidt-Nielsen 1984; Biewener 1989), and combined with resource or space requirements (Carbone *et al*. 2007b), this may ultimately limit maximum body size. In the sea, buoyancy and viscosity influence mobility in relation to body mass, and among vertebrates support much larger species, with maximum sizes among extant mammals about an order of magnitude larger than those on land (Smith & Lyons 2011).

Marine and terrestrial environments also differ in dimensionality, with terrestrial habitats mostly being two-dimensional for non-volant species and marine habitats mainly three-dimensional [for a finer definition of dimensionality see (Pawar *et al*. 2012)”. Dimensionality of habitats may influence animal distributions and rates of interaction (Carbone *et al*. 2007a; Hutchinson & Waser 2007; Pawar *et al*. 2012) because the added dimension provides additional living space (Carbone *et al*. 2007a). These differences in dimensionality may also have important additional impacts on food intake rates, especially on small prey which can be consumed passively and for which intake rate will be limited by the number encountered per unit time. For example, intake rates of invertebrate feeders on land generally only support up to medium-sized predators in the Order Carnivora (Carbone *et al*. 1999). In marine environments, giant predators consume vast numbers of small prey by filtering large volumes of water (Sims 2008; Motta *et al*. 2010; Potvin *et al*. 2012). Clearly, small-prey feeding does not limit predator size in marine environments to the same extent as it does on land, and there is a need for a theoretical framework to develop a broader understanding of how predator diet varies with body size in marine and terrestrial biospheres.

Here, we develop a simple foraging model to predict scaling relationships between predator and minimum prey size, assuming passive harvesting of prey during locomotion, metabolic rate of the predator and the dimensionality of the environment to predict a lower bound for predator–prey size relationships. In combination with the assumption that the upper bound will be related to predator size given the costs and risks of capture and handling (Carbone *et al*. 2007b), our predicted lower limit can be used to predict the prey size range for a given size predator. We test predicted prey ranges empirically using data on diets of 794 terrestrial and marine vertebrate predator species. While vertebrate predators only represent a part of the vast diversity of predatory species, they vary greatly in metabolism (ectotherm and endotherm) and size, and have diets that are reasonably well documented, and thus represent an excellent group to explore variation in predatory strategy.

## Methods

### Geometric foraging model

The foraging model is based on small-prey feeding (e.g. filter-feeding baleen whales) or invertebrate feeding on land (cf. Carbone *et al*. 1999). We assume that catchable small prey are randomly distributed and the predators’ intake rates are proportional to the size of the search path covered in a day, an area defined by the body width (aka reach or mouth width) and the distance travelled. The foraging path width (m) *W* which reflects the immediate area across which prey can be extracted while the predator is moving, is represented as the power function, 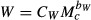, where *M*_*c*_ represents the mass of the predator (c represents ‘consumer’). *C* and *b* represent constants and exponents, with their subscripts relating to the variable name. Daily distance travelled (m/day) *D* is represented by the power function, 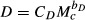 (m/day). On land, the search area *A*_*s*_ (m^2^) is then represented by,

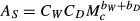
1In a three-dimensional environment, however, the predators cut out a search volume (m^3^), *V*_*s*_, represented by the distance travelled and a frontal body area,

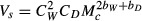
2

We assume an array of different-sized prey species, where biomass (kg/m^2^ on land and kg/m^3^ in the sea) *B* can be represented as a power function of prey mass *M*_*r*_ (r represents ‘resource’)(Peters 1983). We represent daily predator intake rate, *I*_*A*_ (2 dimensions, area) and *I*_*V*_ (3 dimensions, volume) as the product of the search areas or volumes and prey biomass (kg/m^2^ and kg/m^3^ respectively):

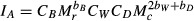
3

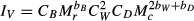
4

Assuming prey biomass is an increasing function of prey mass *M*_*r*_ [i.e. *b*_*B*_ is positive (Damuth 1998; Ernest *et al*. 2003)”, we can predict minimum prey mass required to meet the consumer's resource requirements, *R*_*c*_, where 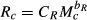, by setting *I*_*A*_ and *I*_*v*_ equal to *R*_*c*_:

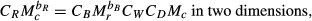
5

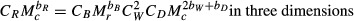
6Solving for *M*_*r*_ gives: in two dimensions,

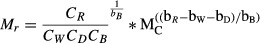
7and in three dimensions:

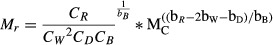
8

**Figure 1:**
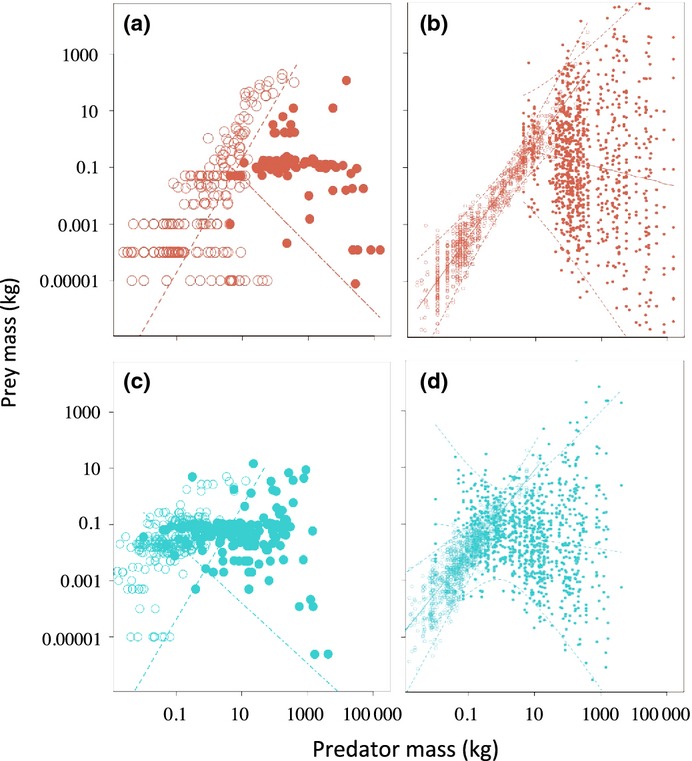
Observed and predicted predator–prey size relationships in (a) mammals (brown points: terrestrial – open circle, marine – filled circle) and (c) ectotherms (blue points: snakes – open circle, elasmobranchs – filled circle). The predicted lower limits in each group [brown and blue dashed lines (terrestrial), dash-dot lines (marine)” were estimated from eqns 7 and 8, using constants and exponents given in Table1. Predicted spread in prey sizes were generated by the two- and three-dimensional models using the observed predator mass and randomising mean parameters in Table1 by ± 12.5%; (b) model mammal predators (brown; terrestrial – open circle; marine – filled circle) and (d) model snake and shark predators (blue; terrestrial – open circle; marine – filled circle).

If there is no scaling of prey biomass (i.e. *b*_*B*_ = 0), one can estimate the predator mass supported on a given biomass density (Appendix S3). A number of these parameters may vary across taxa and environments. Table1 provides values for some taxon-specific parameters while using common values where possible to focus on broad-scale relationships. Metabolic rate estimates are based on intake rate estimates of different representative species initially assuming a universal scaling exponent of 0.75, given this has been estimated as the modal exponent for metabolism across a wide range of taxonomic groups (Isaac & Carbone 2010) (but see below). For snakes, the metabolic scaling constant was estimated as approximately 1/10 that of mammals (Peters 1983). In elasmobranchs, we used estimates of intake rates from field studies (Sims 2000, 2008). We also used the same constant and exponent for body width *C*_*w*_ and *b*_*w*_ for all predators, based on estimates of filter area in baleen whales (Nemoto & Kawamura 1977). For terrestrial predators, we use an estimate for the scaling of daily distance moved of 0 (i.e. we estimate no significant variation in speed across predators of different size). This differs from previous broad-scale studies on mammals across dietary groups (Jetz *et al*. 2004; Carbone *et al*. 2006) but is consistent with the variation in speed and intake rate found in invertebrate feeding mammalian carnivores (Carbone *et al*. 1999) (see also below). In practice, terrestrial invertebrate intake rates may reflect limits in the scaling of both speed and foraging path width (the latter was not measured by Carbone *et al*. 1999). We also explore the model predictions with a positive scaling of daily distance moved for terrestrial mammals and snakes (see Fig.1 and Table1). Estimates of the intercepts of the scaling of speed *C*_*D*_ for terrestrial mammals and snakes were obtained from references in Appendix S1, with higher values for mammals than snakes possibly linked with differences in metabolism and body temperature (Bennett & Ruben 1979). The scaling of swim speeds for whales and elasmobranchs were estimated with intercepts calibrated from marine predator swim speeds (Hedenström 2003). Using the above parameter estimates, we predict minimum prey size to scale positively with terrestrial predator mass^2.0^ (eqn 7) but negatively with respect to marine predator mass^−1.2^ (eqn 8).

**Table 1:** The parameters used for the predicted predator–prey relationships in Fig. 1; parameters represent constants and exponents of allometric power equations with the form Y = C^*^M^*b*^

		Metabolic rate (kg/day)		Foraging path width (m)		Predator daily distance moved (m)		Prey biomass (kg/m^2^, terrestrial or kg/m^3^, marine	
Biome	Taxonomic group	*C*_*R*_	*b*_*R*_	*C*_*W*_	*b*_*W*_	*C*_*D*_	*b*_*D*_	*C*_*B*_	*b*_*B*_
Universal	All taxa	–	**0.75**	**0.0423**	**0.349**	–	–	–	–
Terrestrial	mammals	**0.171**	(0.77)			**537** (350)	**0** (0.10)	**0.026**	**0.20**
	snakes	**0.017**	(0.889)			**45.2** (20)	**0** (0.25)		
Marine	mammals	**0.171**	(0.77)			**12532**	**0.17** (0.10)	**0.008**	**0.10**
	sharks	**0.052**	(0.84)			**6800** (5000)	**0.16**		

Values used for lower prey size thresholds in Fig.1a and c are in bold text. Universal values were used where possible (see text for details). Parameter values used for predicting the estimated spread of predator–prey relationships, Fig.1b and d, were randomly varied ± 12.5%; mean values are shown in parentheses. References and further details are given in Appendix S1.

### Predator–prey body masses

We collated predator masses for mammals (Jones *et al*. 2009), snakes (Feldman & Meiri 2013) and elasmobranchs (Froese & Pauly 2014). Body masses of prey were based on broad diet descriptions (cf. Carbone *et al*. 1999). For small prey, where species-specific weights were not given, mass was approximated according to the broad categories, ranging between 0.000008 and 0.22 kg for aquatic invertebrates; 0.00005 to 0.07 kg for terrestrial invertebrates; 0.02 to 0.5 kg for small vertebrates (Appendix S2). In elasmobranchs and marine mammals, we either used individual published studies of diet or used two comparative studies focusing on the trophic levels and diets of elasmobranchs and marine mammals (Pauly *et al*. 1998; Cortés 1999). In these compilations of marine predator diets, categories needed to be converted into mass classes using references in Appendix S1.

We used ordinary least squares (OLS) and phylogenetic generalised least squares (PGLS) to test how prey mass changed as a function of predator mass, estimating the phylogenetic signal (λ) with maximum likelihood (Revell 2010). Phylogenetic trees were created in TreeView (Page 1996), and where species relationships were not fully resolved within the genus, these were coded as a polytomy. We pruned a supertree of extant mammals (Bininda-Emonds *et al*. 2007) for the terrestrial mammal predators; for the other groups, trees were composed from various sources (Appendix S1). Because information on branch length was not available for non-mammal clades, branch lengths were set to 1. For mammals, analyses were conducted both using actual branch lengths and branch lengths set to 1.

Statistical tests were performed in R 2.15.0 (R Core Development Team) using the package *caper* (Orme *et al*. 2010). In contrast to a common recommendation for comparative studies (Freckleton 2009), we display results of both OLS and PGLS analyses, because discrepancies between the two can yield important insight into the data patterns (Clauss *et al*. 2014). Among terrestrial predators, based on model predictions, we expected minimum prey size, which scales greater than predator mass (model prediction) and the upper bound (which we expect to scale with mass) to converge as predator size increases and so expect significant positive predator–prey relationships. In marine environments, on the other hand, due to the fact that minimum and maximum prey size diverge with increasing predator size, we expected to find only weak negative relationships.

We also used the 2D and 3D models to predict prey size ranges for the same predator masses for each group, by randomly sampling (10000 times) mean species-specific parameters (Table1) with a uniform distribution ± 12.5%. This was done to simulate natural variation in scaling parameters which we assume occurs across taxonomic groups. We can then assess whether the variation in the model predictions can be used to predict observed variation in prey sizes taken across predator groups.

## Results

There was a significant phylogenetic signal in all four data sets of this study (λ ≠ 0, Table2). Overall, relative sizes of predators and prey match the broad qualitative scaling predictions of the model (Fig.1). Among terrestrial predators, prey size increased significantly with predator size, but at a higher level in snakes (overlapping 95% CI for the exponent but not for the scaling factor in Table2; Fig.1). Results were qualitatively similar in OLS and PGLS. The mammal scaling exponent was distinctly lower when all branch lengths were set to 1 as compared to when actual branch lengths were used (Table2).

**Table 2:** Result of comparative analyses of how minimum prey size varies as a function of predator size

Taxonomic group/biome	*n*	Body mass (kg, mode, range)		Stat	λ*	a (95% CI)	*t*	*P*	b (95% CI)	*t*	*P*
		Predator	Prey								
Terrestrial mammals	270	0.112 (0.002–371)	0.0001 (0.000001–189)	OLS	(0)	0.007 (0.004; 0.010)	−22.456	0.000	1.05 (0.90; 1.20)	13.709	0.000
				PGLS§	0.929‡	0.0003 (0.00001; 0.013)	−4.276	0.000	0.82 (0.60; 1.03)	7.381	0.000
				PGLS¶	1.0†	0.0001 (0.00001; 0.001)	−7.923	0.000	0.36 (0.15; 0.57)	3.293	0.001
Marine Mammals	126	23000 (4–154160)	0.100 (0.00003–12)	OLS	(0)	0.546 (0.215; 1.386)	−1.274	0.205	−0.30 (−0.45; −0.15)	−3.975	0.000
				PGLS¶	0.978†	0.013 (0.001; 0.232)	−2.940	0.004	0.16 (−0.13; 0.44)	1.054	0.294
Snakes	228	0.037 (0.0006–13)	0.035 (0.000005–2.6)	OLS	(0)	0.249 (0.151; 0.412)	−5.416	0.000	0.88 (0.72; 1.03)	11.176	0.000
				PGLS¶	1.0†	0.008 (0.001; 0.069)	−4.349	0.000	0.68 (0.52; 0.84)	8.340	0.000
Elasmobranchs	168	16.4 (0.014–4250)	0.005 (0.0000006–15)	OLS	(0)	0.051 (0.033; 0.077)	−13.866	0.000	−0.09 (−0.22; 0.04)	−1.374	0.171
				PGLS¶	1.0†	0.011 (0.001; 0.143)	−3.455	0.001	0.05 (−0.11; 0.21)	0.648	0.518

Parameter estimates, 95% CIs, *t*-statistics and *P*-values are given for *a* and *b* in the equation prey size = a^*^predator size^*b*^, for terrestrial mammals; snakes; marine mammals; elasmobranchs performed with ordinary least squares (OLS) and phylogenetic generalised least squares (PGLS).

* λ estimated by maximum likelihood.

† λ significantly different from 0.

‡ λ significantly different from 0 and 1.

§ phylogenetic tree with actual branch lengths.

¶ phylogenetic tree with branch lengths set to 1.

Among marine predators, the breadth of prey sizes increased visually with predator size (Fig.1). When we randomly vary all model parameters ± 12.5%, the 2D and 3D models produced similar patterns of variation in predator–prey relationships to the observed patterns in terrestrial and marine predators (Fig.1b and d).

For marine predators, OLS yielded a significant negative scaling for mammals but no significant relationship in elasmobranchs (Table2). The PGLS result resembled that of OLS in elasmobranchs, indicating that also among closely related species, there is no scaling effect for prey size. For marine mammals, the PGLS was non-significant (Table2).

**Figure 2:**
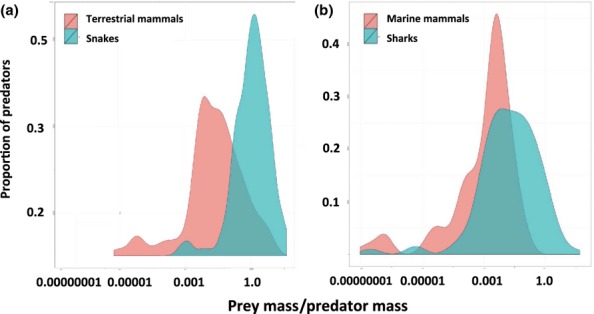
Observed proportion of prey–predator mass ratios organised by group: (a) terrestrial mammals (brown shading), snakes (blue shading); (b) marine mammals (brown), sharks (blue).

Most predators take relatively small prey, with approximately 60% of species taking prey ≤ 1/100 of their weight. Modal prey size classes by all predatory groups ranged from 0.1 to 100 g (Table2). Elasmobranchs were roughly an order of magnitude smaller than marine mammals on similar diets (larger prey-predator size ratio); similarly, snakes selected relatively larger prey than similar-sized terrestrial mammals (larger prey–predator size ratio) (Table2, Fig.2). A number of larger terrestrial mammal predators specialise on small prey, which was not predicted by our model (Fig.1a).

## Discussion

Our results highlight differences in predatory strategies between terrestrial and marine environments. We examined the empirical patterns in relation to the predictions of our foraging model. Consistent with the predictions, we found significant positive relationships between predator and prey size among terrestrial predators using both OLS and PGLS, indicating that this relationship is convergent across taxonomic subgroups (Table2). For mammals, our results are consistent with those of a very recent study based on a different data collection (Tucker & Rogers 2014).

In marine environments, the allometric relationships between search volume and predator mass were predicted to facilitate small-prey feeding among the largest predators, and we predicted a weak negative relationship in these groups. The observed relationships between predator and average prey size were significantly negative in OLS only in the marine mammals but not in elasmobranchs, and were not significant in PGLS for either group. The largest marine predators (whale sharks and baleen whales) are filter feeders, feeding mostly on krill (Sims 2008; Motta *et al*. 2010; Potvin *et al*. 2012). However, some large marine predators have broad diets including large vertebrate prey, [e.g. great white shark *Carcharodon carcharias* (Estrada *et al*. 2006) and orca *Orcinus orca* (Ford *et al*. 1998)”. The difference in the OLS result between sharks and mammals reflects the higher species diversity in filter feeding among whales and the higher species diversity in sharks feeding on larger prey; the difference between the OLS and PGLS results within the marine mammals indicates that there are not multiple taxonomic subgroups in which the relationship can be observed, but that small-prey (filter) feeding evolved only once (Slater *et al*. 2010).

Interestingly, predicted prey sizes produced from varying the model parameters ± 12.5% were similar to patterns observed among terrestrial and marine predators (Fig.1). This process allows us to mimic the likely variation in scaling exponents and constants that may occur at finer taxonomic levels (Isaac & Carbone 2010; Isaac *et al*. 2011). The differences in the two models’ sensitivity to variation in parameters are due to the influence of scaling of speed *b*_*D*_ and path width *b*_*w*_ relative to the scaling of metabolism *b*_*R*_ (Appendix S3). In the 3D model, the added dimension resulting in the term 2*b*_*w*_ means that lower values of *b*_*w*_ or *b*_*D*_ can result in the model switching from a negative to a positive scaling. Among the marine predators, the baleen whales are clearly highly adapted for maximising *b*_*w*_ when lunging for prey (Goldbogen *et al*. 2010). However, further work is needed to understand the relative constraints on prey selection in relation to size and metabolism among marine predators, although it appears there is great potential for diversity in these systems. While the model predicts similar patterns in diet breadth among marine predators, it does not predict the strong predominance of prey between 35 and 100 g (Fig.1; Table2), which may reflect a peak in biomass density of prey species present in marine environments (Koslow *et al*. 1997; Pauly *et al*. 1998).

In contrast, the model predicts a robust positive scaling among terrestrial predator diets, which is consistent with the observed patterns (Fig.1a and b). In mammals, however, the model does not predict the observed diversity of medium–large terrestrial predators feeding on small invertebrates (Fig.1a). This pattern was not found in snakes. This is perhaps surprising; given snakes have lower metabolism, they should be able to persist on smaller prey. However, snakes exhibit lower mobility and overall movement rates probably also linked with metabolism (Appendix S1); given this reduced mobility, they appear to be forced to feed on more substantial vertebrate prey at smaller sizes than mammals. These findings lead to the hypothesis that endothermy, through higher mobility, generally facilitates insectivory at larger body sizes than ectothermy. However, larger mammal invertebrate feeders are also highly specialised and may enhance intake rates by focussing on social invertebrates, and alleviate metabolic constraints by maintaining low metabolic rates (Stahl *et al*. 2012).

Overall, we found that ectotherms take relatively larger prey than endotherms in both terrestrial and marine environments, although possibly for different reasons. Elasmobranchs with their lower metabolism can maintain sufficient intakes on a given prey size at a smaller size relative to marine mammals. Snakes (as discussed above) may select relatively large prey because of reduced mobility.

Our model is based on simple geometric assumptions. It does not include many factors such as detection or reaction distance, handling constraints or functional responses (McGill & Mittelbach 2006; Pawar *et al*. 2012). As such, it ignores many of the complicated behaviours associated with the diverse types of predators included in this study. Prey mobility may be a factor particularly important for sit-and-wait strategies, but is not accounted for in our model. The model assumptions may be less suitable for predators feeding on large highly mobile prey. However, 60% of the predators in this study predominantly feed on small prey (≤ 1/100 of predator size). Models which consider detection distance may be particularly suited for predators with high sensual acuity, such as vision or smell (Pawar *et al*. 2012). Our model complements that of Pawar *et al*. (2012) by focusing on encounter-rate-based foraging strategies. In the future, the model could be adapted to accommodate prey movement rates in addition to predator movement rates to explore how this factor affects prey size selection.

Despite its simplicity, our model makes novel predictions. Large marine filter feeders are often described as special cases, which are sometimes excluded from comparative analyses (Cortés 1999; Brose *et al*. 2006). Studies focused on rorqual whales describe the inverse scaling of prey size in this group as resulting from larger species specialising on krill instead of fish due to reduced mobility (Goldbogen *et al*. 2010). This explanation, although sensible, does not explain the absence of filter feeders among smaller aquatic vertebrate predators. Our model puts extremely diverse predator species into a broad theoretical context.

The predictions are sensitive to model parameters, which are in some instances poorly estimated and may vary between taxonomic levels. In Appendix S3, we describe how the model varies within reasonable parameter ranges. For Table1, where possible, we have adopted a strategy to use broad-scale estimates of parameters across groups. However, while further research is needed to improve our estimates of some of the parameters in the model, there is considerable value in this approach for understanding qualitative patterns. Future research could explore smaller scale patterns using case-specific parameters and conditions where these may be better defined.

One important limitation of this study is our lack of understanding of prey species abundance on global scales. This is particularly important for predictions that depend on the scaling of standing biomass density. In terrestrial systems, biomass density is widely found to scale positively with body mass, around mass^0.25^; however, in marine environments where larger species occur at higher trophic levels, the scaling of biomass and abundance may be negative (Cohen *et al*. 2003; Blanchard *et al*. 2009). Indeed, our finding that there is a predominance of marine prey in the 0.1–100 g size range suggests at least a partial deviation from simple scaling of prey biomass in marine systems. If the biomass density in marine ecosystems does not scale with prey body mass, then conditions for our estimate of predator–prey mass would not be met, but our approach could be modified to predict a predator mass threshold for a specific prey biomass density (Appendix S3).

Our prey mass estimates range from an average estimate of the masses of common prey types, to a broad characterisation of diet represented by a mass estimate. Predator weights in indeterminate growth species (e.g. sharks and snakes) may vary greatly across individuals within a species. Ideally, in such species one would use diet data from individuals of known weight (e.g. King 2002). Our estimates of prey–predator ratios are sensitive to error in weights. While error in our data will influence individual values, we are confident, given the vast size ranges over which our patterns are observed, that our main findings will not be biased by these levels of error.

Our study focuses on large vertebrate predators and the predictions are based on metabolic rates and movement rates of these species; for smaller predators with lower metabolic rates (e.g. smaller fish) or species living in food rich environments, we would expect limits on small-prey feeding to be lower. Considering more diverse taxa such as groups of invertebrates, differences in size, metabolism and the scaling of mobility would have a critical influence on the model predictions and these may differ for these groups. For example, the three-dimensional model predicts positive scaling in predator–prey size if travelling speed or path width does not scale with mass. In contrast to vertebrates, evidence for positive scaling of swim speed in invertebrates is equivocal (McHenry 2003; Chan *et al*. 2013).

An additional factor we have not considered is the influence of environmental temperature on predator ecology, which if related to body temperature (e.g. ectotherms) may have an important affect on predator strategy. While our model does not include temperature as a specific parameter, if temperature affects metabolic rate constants *C*_*R*_ and speed *C*_*D*_ (Bennett & Ruben 1979), these factors could be included in the model to predict changes in predator–prey relationships in response to changes in environmental temperature. This illustrates that our approach could be easily adapted to address a wide range of issues including possible influences of climate change on ecosystem function.

Although simple, our model provides novel insights concerning the influence of environment and predator metabolism on predator–prey relationships, and as such has important implications for our understanding of terrestrial and marine systems.
