# Supplementary material for: Geometric factors influencing the diet of vertebrate predators in marine and terrestrial environments
Source: Ecol Lett. 2014 Sep 30;17(12):1553–9. doi: 10.1111/ele.12375 (PMC4284001; doi:10.1111/ele.12375)
Supplement: Supplementary file 1 [file ele0017-1553-sd1.docx]

Appendix 1 Additional References:

1. References used in Table 1, see text for details.

| Biome | Taxonomic group | Metabolic rate (kg/day) | | Foraging path width (m) | | Predator daily distance moved (m) | | Prey biomass (kg/m^2^, terrestrial or kg/m^3^, marine | |
| --- | --- | --- | --- | --- | --- | --- | --- | --- | --- |
|  |  | C_R_ | b_R_ | C_W_ | b_W_ | C_D_ | b_D_ | C_B_ | b_B_ |
| Universal | All taxa | - | **0.75**  [1] | **0.0423**  [2, 3] | **0.349**  [2, 3] |  |  |  |  |
| Terrestrial | mammals | **0.171**  [2] | (0.77)  [4] |  |  | **537** (350)* | **0** (0.10) | **0.026**  [5] | **0.20**  [5] |
|  | snakes | **0.017**  [6] | (0.889)  [4] |  |  | **45.2** (20)*  [7-15] | **0** (0.25) |  |  |
| Marine | mammals | **0.171**  [2] | (0.77)  [4] |  |  | **12532**  [16] | **0.17** (0.10) | **0.008**  [17, 18] | **0.10**  [19-21] |
|  | sharks | **0.052**  [17, 22] | (0.84)  [22] |  |  | **6800** (5000)* | **0.16** |  |  |

* – values modified for predicted patterns in Figure 1b & d, but these constants are within the range found [23, 24] and herpetological references below.

2) References for Phylogenies:

marine mammals: [25-28]

snakes [29, 30];

elasmobranchs [31, 32]

3) References for estimating marine prey mass classes:

[33-40]

Reference List:

1. Isaac, N.J.B. and C. Carbone, *Why are metabolic scaling exponents so controversial? Quantifying variance and testing hypotheses.* Ecology Letters, 2010. **13**: p. 728-735.

2. Carbone, C., A. Teacher, and J.M. Rowcliffe, *The costs of carnivory.* PLoS Biology, 2007. **5**: p. e22.

3. Nemoto, T. and A. Kawamura, *Characteristics of food habits and distribution of baleen whales with special reference to the abundance of North Pacific sei and Bryde’s whales.* Reports of the International Whaling Commission, 1977. **Special 1**: p. 80-87.

4. Nagy, K.A., I.A. Girard, and T.K. Brown, *Energetics of free-ranging mammals, reptiles, and birds.* Annual Review of Nutrition, 1999. **19**: p. 247-277.

5. Carbone, C., et al., *Energetic constraints on the diet of terrestrial carnivores.* Nature, 1999. **402**: p. 286-288.

6. Peters, R.H., *The ecological implications of body size*. 1983, Cambridge: Cambridge University Press.

7. Gent, A.H. and I.F. Spellerberg, *Movement rates of the smooth snake Coronella austriaca (Colubridae): a radio-telemetric study.* Herpetological Journal, 1993. **3**: p. 140-146.

8. Secor, S.M., *Ecological significance of movements and activity range for the sidewinder (Crotalus cerastes).* Copeia, 1994. **1994**: p. 631-645.

9. Wilson, D., R. Heinsohn, and S. Legge, *Age‐and sex‐related differences in the spatial ecology of a dichromatic tropical python (Morelia viridis).* Australian Ecology, 2006. **31**: p. 577-587.

10. Lee, H.J., J.H. Lee, and D. Park, *Habitat use and movement patterns of the viviparous aquatic snake (Oocatochus rufodorsatus) from Northeast Asia.* Zoological Science, 2011. **28**: p. 593-599.

11. Plummer, M.V. and J.D. Congdon, *Radiotelemetric study of activity and movements of racers (Coluber constrictor) associated with a Carolina bay in South Carolina.* Copeia, 1994. **1994**: p. 20-26.

12. Beck, D.D., *Ecology and energetics of three sympatric rattlesnake species in the Sonoran Desert.* Journal of Herpetology, 1995. **29**: p. 211-223.

13. Keogh, J.S., J.K. Webb, and R. Shine, *Spatial genetic analysis and long-term mark–recapture data demonstrate male-biased dispersal in a snake.* Biology Letters, 2007. **3**: p. 33-35.

14. Webb, J.K. and R. Shine, *A field study of spatial ecology and movements of a threatened snake species (Hoplocephalus bungaroides).* Biological Conservation, 1997. **82**: p. 203-217.

15. Wasko, D.K. and M. Sasa, *Activity patterns of a neotropical ambush predator: Spatial ecology of the Fer‐de‐lance (Bothrops asper, Serpentes: Viperidae) in Costa Rica.* Biotropica, 2009. **41**: p. 241-249.

16. Hedenström, A., *Scaling migration speed in animals that run, swim and fly.* Journal of Zoology, 2003. **259**: p. 155-160.

17. Sims, D.W., *Sieving a living: A review of the biology, ecology and conservation status of the plankton‐feeding basking shark (Cetorhinus maximus).* Advances in Marine Biology, 2008. **54**: p. 171-220.

18. Motta, P.J., et al., *Feeding anatomy, filter-feeding rate, and diet of whale sharks (Rhincodon typus) during surface ram filter feeding off the Yucatan Peninsula, Mexico.* Zoology, 2010. **113**: p. 199-212.

19. Koslow, J.A., R.J. Kloser, and A. Williams, *Pelagic biomass and community structure over the mid-continental slope off southeastern Australia based upon acoustic and midwater trawl sampling.* Marine Ecology Progress Series, 1997. **146**: p. 21-35.

20. Jennings, S., et al., *Global-scale predictions of community and ecosystem properties from simple ecological theory.* Proceedings of the Royal Society B, 2008. **275**: p. 1375-1383.

21. Gasol, J.M., P.A. del Giorgio, and C.M. Duarte, *Biomass distribution in marine planktonic communities.* Limnology and Oceanography, 1997. **42**: p. 1353-1363.

22. Sims, D.W., *Can threshold foraging responses of basking sharks be used to estimate their metabolic rate?* Marine Ecology Progress Series, 2000. **200**: p. 289-296.

23. Carbone, C., et al., *How far do animals go? Determinants of day range in mammals.* American Naturalist, 2006. **165**: p. 290-297.

24. Garland, T., *The relation between maximal running speed and body mass in terrestrial mammals.* Journal of Zoology, 1983. **199**(2): p. 157-170.

25. Bininda-Emonds, O.R.P., et al., *The delayed rise of present-day mammals.* Nature, 2007. **446**: p. 507-512.

26. Koepfli, K.P. and R.K. Wayne, *Phylogenetic relationships of otters (Carnivora: Mustelidae) based on mitochondrial cytochrome b sequences.* Journal of Zoology, 1998. **246**: p. 401-416.

27. Higdon, J.W., et al., *Phylogeny and divergence of the pinnipeds (Carnivora: Mammalia) assessed using a multigene dataset.* BMC Evolutionary Biology, 2007. **7**: p. 216.

28. Price, S.A., O.R.P. Bininda-Emonds, and J.L. Gittleman, *A complete phylogeny of the whales, dolphins and even-toed hoofed mammals (Cetartiodactyla).* Biological Reviews, 2005. **80**: p. 445-473.

29. Wiens, J.J., et al., *Branch lengths, support, and congruence: testing the phylogenomic approach with 20 nuclear loci in snakes.* Systematic Biology, 2008. **57**: p. 420-431.

30. Pyron, R.A., et al., *The phylogeny of advanced snakes (Colubroidea), with discovery of a new subfamily and comparison of support methods for likelihood trees.* Molecular Phylogenetics and Evolution, 2011. **58**: p. 329-342.

31. Vélez-Zuazo, X. and I. Agnarsson, *Shark tales: a molecular species-level phylogeny of sharks (Selachimorpha, Chondrichthyes).* Molecular Phylogenetics and Evolution, 2011. **58**: p. 207-217.

32. Winchell, C.J., A.P. Martin, and J. Mallatt, *Phylogeny of elasmobranchs based on LSU and SSU ribosomal RNA genes.* Molecular phylogenetics and evolution, 2004. **31**(1): p. 214-24.

33. Belcari, P., *Length-weight relationships in relation to sexual maturation of Illex coindetii (Cephalopoda: Ommastrephidae) in the norther Tyrrhenian Sea (Western Mediterranean).* Scientia Marina, 1996. **60**(2-3): p. 379-384.

34. Benoit-Bird, K.J., et al., *Dusky dolphin (Lagenorhynchus obscurus) foraging in two different habitats: Active acoustic detection of dolphins and they prey.* Marine Mammal Science, 2004. **20**: p. 215-231.

35. Dunham, J.S. and D.A. Duffus, *DIET OF GRAY WHALES (ESCHRICHTIUS ROB USTUS) IN CLAYOQUOT SOUND, BRITISH COLUMBIA, CANADA.* Marine Mammal Science, 2002. **18**(April): p. 419-437.

36. Hofmann, E. and C. Lascara, *Modeling the growth dynamics of Antarctic krill Euphausia superba.* Marine Ecology Progress Series, 2000. **194**: p. 219-231.

37. Motta, P.J., et al., *Feeding anatomy, filter-feeding rate, and diet of whale sharks Rhincodon typus during surface ram filter feeding off the Yucatan Peninsula, Mexico.* Zoology, 2010. **113**(4): p. 199-212.

38. Potier, M., et al., *Length and weight estimates from diagnostic hard part structures of fish, crustacea and cephalopods forage species in the western Indian Ocean.* Environmental Biology of Fishes, 2011: p. 1-11-11.

39. Santos, R.A.d. and M. Haimovici, *Cephalopods in the diet of marine mammals stranded or incidentally caught along southeastern and southern Brazil (21–34°S).* Fisheries Research, 2001. **52**(1-2): p. 99-112.

40. Sims, D.W., *Sieving a living: a review of the biology, ecology and conservation status of the plankton-feeding basking shark Cetorhinus maximus.* Advances in marine biology, 2008. **54**(08): p. 171-220.
