# Supplementary material for: Geometric factors influencing the diet of vertebrate predators in marine and terrestrial environments
Source: Ecol Lett. 2014 Sep 30;17(12):1553–9. doi: 10.1111/ele.12375 (PMC4284001; doi:10.1111/ele.12375)
Supplement: Supplementary file 3 [file ele0017-1553-sd3.docx]

1. Scenarios without biomass density scaling:

The derivations in equations 7 and 8, assume that *b_B_* ≠ 0. If there is no overall scaling in prey biomass density, one can estimate a limit to predator size from equations 5 and 6 based on estimating sustainable intake rate, below (see text for details):

$C_{R}{M_{c}}^{b_{R}}=C_{B}{M_{r}^{b_{B}} C}_{W}C_{D}{M_{c}}^{b_{W}{+ b}_{D}}$ in two dimensions and (eq. 5)

$C_{R}{M_{c}}^{b_{R}}=C_{B}M_{r}^{b_{B}}{C_{W}}^{2}C_{D}{M_{c}}^{{2b}_{W}{+ b}_{D}}$ in three dimensions (eq. 6).

Without prey biomass scaling we remove $M_{r}^{b_{B}}$ term,

$C_{R}{M_{c}}^{b_{R}}=C_{B}C_{W}C_{D}{M_{c}}^{b_{W}{+ b}_{D}}$ in two dimensions and

$C_{R}{M_{c}}^{b_{R}}=C_{B}{C_{W}}^{2}C_{D}{M_{c}}^{{2b}_{W}{+ b}_{D}}$ in three dimensions.

We can then solve for $M_{c}$ as follows:

$M_{c}= {(\frac{C_{B}C_{W}C_{D}}{C_{R}})}^{\frac{1}{{b_{R}-(b}_{W}{+ b}_{D})}}$

$M_{c}= {(\frac{C_{B}{C_{W}}^{2}C_{D}}{C_{R}})}^{\frac{1}{{b_{R}-(2b}_{W}{+ b}_{D})}}$

2. Model Sensitivity

$M_{R}=\left. {\frac{C_{R}}{C_{W}C_{D}C_{B}}}^{\frac{1}{b_{B}}} * M_{C}^{\left( {(b}_{R}- b_{W}{- b}_{D})/b_{B} \right)} \right.$ (7)

and in three dimensions:

$M_{R}={\frac{C_{R}}{{C_{W}}^{2}C_{D}C_{B}}}^{\frac{1}{b_{B}}} * M_{C}^{\left( {(b}_{R}- {2b}_{W}{- b}_{D})/b_{B} \right)}$ (8)

In equations 7 and 8 above it is clear that $b_{B}$ has a large influence on the model as it appears in both the exponent and constant terms (Figure S1) and the effects of *b_B_* increase as it approaches 0. Moderate deviations from the estimates in Table 1, produce moderate changes in the predicted constant and exponent terms. For example a ±12.5% change in *b_B_* results in a change in the exponent (${}^{\left( {(b}_{R}- {2b}_{W}{- b}_{D})/b_{B} \right)}$) term by 11.1 to 14.3% respectively. A ±30% change in this variable results in a 23.1 to 42.9% change respectively. This same level of variation in *b_B_* for the constant term produces a smaller degree of variation ranging from 2.4% (for 12.5% variation in *b_B_*) and a maximum of 12.2% given a 30% change in *b_B_*.

Exponents $b_{R}, b_{W}{, b}_{D}$ all have equal effect on the exponent term as these are additive terms in the two-dimensional model, However, in the three-dimensional model $b_{W}$ has double the influence of the other exponent terms. An important feature of the model is that the scaling is dependent on the relative values of the metabolic scaling exponent *b_R_* and the other exponent terms. The added dimension (*b_W_*) in three-dimensional environments creates a scenario where the scaling of minimum mass is negative. However, Fig 1b and 1d, show that the three-dimensional model is sensitive to variation in these parameters and can lead to predictions of positive scaling under some scenarios (see text for details).

In Figure S1, the overall influence of *b_B_* on predicted *M_R_* is shown. *b_B_* has a strong affect on *M_R_* at low values which increases the scaling (seen here as increasing gaps between predator mass classes). However, in practice, when fitting power equations to real animal assemblages, the slope and intercept vary together and in this instance, lowering *b_B_* would lead to a lowering of *C_B_* (not shown here), and this would have had a moderating effect on the variation of *M_r_*. The natural log value of minimum prey size found in this study is around 11.5 (see Figure S1 below).


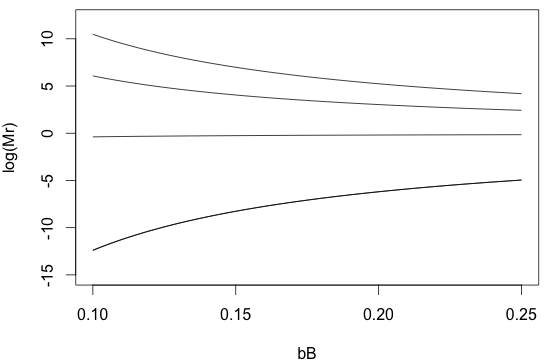

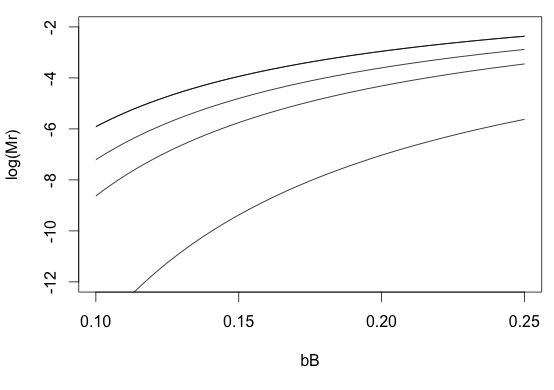


2D

3D

**20**

**1**

**300**

**20**

**100**

**100**

**300**

**1000**

Figure S1. The affect of *b_B_* on predicted M*_R_* for the two and three dimensional models (see text for details) estimated using values for the remaining parameter estimates given in Table 1 in the text for mammals (terrestrial and marine).
